# Management of Progressive Fibrosing Interstitial Lung Diseases (PF-ILD)

**DOI:** 10.3389/fmed.2021.743977

**Published:** 2021-10-13

**Authors:** Carla R. Copeland, Lisa H. Lancaster

**Affiliations:** Department of Medicine, Vanderbilt University Medical Center, Nashville, TN, United States

**Keywords:** interstitial lung disease, pulmonary fibrosis, progressive fibrosing interstitial lung disease, idiopathic pulmonary fibrosis, antifibrotics

## Abstract

Progressive fibrosing interstitial lung diseases (PF-ILD) consist of a diverse group of interstitial lung diseases (ILD) characterized by a similar clinical phenotype of accelerated respiratory failure, frequent disease exacerbation and earlier mortality. Regardless of underlying disease process, PF-ILD progresses through similar mechanisms of self-sustained dysregulated cell repair, fibroblast proliferation and alveolar dysfunction that can be therapeutically targeted. Antifibrotic therapy with nintedanib or pirfenidone slow lung function decline and are the backbone of treatment for IPF with an expanded indication of PF-ILD for nintedanib. Immunosuppression is utilized for some subtypes of PF-ILD, including connective tissue disease ILD and hypersensitivity pneumonitis. Inhaled treprostinil is a novel therapy that improves exercise tolerance in individuals with PF-ILD and concomitant World Health Organization (WHO) group 3 pulmonary hypertension. Lung transplantation is the only curative therapy and can be considered in an appropriate and interested patient. Supportive care, oxygen therapy when appropriate, and treatment of comorbid conditions are important aspects of PF-ILD management. This review summarizes the current data and recommendations for management of PF-ILD.

## Introduction

Idiopathic pulmonary fibrosis (IPF) is the archetypal progressive fibrotic interstitial lung disease (ILD) characterized by accelerated respiratory failure, frequent disease exacerbation and earlier mortality (1, 2). Despite generally better outcomes with non-IPF fibrotic ILD, some individuals develop a progressive phenotype similar to IPF (3, 4). This progressive fibrosing interstitial lung disease (PF-ILD) phenotype is seen with connective tissue disease (e.g., rheumatoid arthritis, scleroderma, dermatomyositis/polymyositis) associated ILD (CTD-ILD), fibrotic hypersensitivity pneumonitis (fHP), pneumoconioses (e.g., asbestosis, silicosis), sarcoidosis, idiopathic non-specific interstitial pneumonia (NSIP), and unclassifiable ILD (3, 5, 6). Risk factors for progression include older age, male sex, lower baseline pulmonary function, and radiographic honeycombing or usual interstitial pneumonia (UIP) pattern of injury (7, 8). Regardless of disease trigger, PF-ILD progresses through mechanisms of self-sustained dysregulated cell repair, fibroblast proliferation and alveolar dysfunction that can be targeted similarly (5, 6, 9). This review summarizes our current understanding of pharmacologic and non-pharmacologic treatment options for PF-ILD.

## Pharmacologic Therapies

### Antifibrotic Therapies

Nintedanib is an oral intracellular tyrosine kinase inhibitor that blocks cell-signaling pathways involved in fibrosis progression (10, 11). At a dose of 150 mg twice daily, nintedanib reduces the annual decline in forced vital capacity (FVC) in individuals with IPF (11), scleroderma associated pulmonary fibrosis (12), and, more recently, non-IPF progressive pulmonary fibrosis (9). In the randomized controlled *INBUILD* trial, the majority of individuals had a usual interstitial pneumonia (UIP) imaging pattern and were diagnosed with fibrotic HP, CTD-ILD, idiopathic NSIP, or unclassifiable ILD. All participants had progressive disease defined as a 10% FVC decline, a ≥5% FVC decline with symptom or imaging progression, or worsening symptoms and imaging (9). With nintedanib the adjusted yearly FVC change was −80.8 ml compared to −187.8 ml with placebo (between-group difference 107.0 ml, 95%CI 65.4–148.5; *P* < 0.001) (9), similar to efficacy in IPF (11).

Diarrhea is the most common side effect with nintedanib, occurring in 60–76% of individuals (9, 11, 12). Anti-diarrheal agents included in the drug blister pack can be utilized as needed (2, 11). Elevations in alanine aminotransferase (ALT) and aspartate aminotransferase (AST) can occur, though only about 5% of patients have significant elevations (e.g., ≥ three times upper limit of normal) (9, 11, 12). Liver function tests (LFTs) should be monitored monthly for the first 3 months and then every 3–4 months while on treatment (2). If side effects occur, concomitant drugs and infections should be excluded and dose reduction to 100 mg twice daily or a dosing holiday can be trialed; however, approximately 20% of individuals may still discontinue the drug due to intolerable side effects (9, 11, 12).

Pirfenidone is another oral antifibrotic agent that reduces fibrotic progression by inhibition of collagen synthesis and fibroblast proliferation (13). Pirfenidone slows the FVC decline in IPF (14) and unclassifiable progressive fibrotic ILD (15). Notably, the latter study did not meet its primary endpoint of home spirometry due to unforeseen measurement variability and utilized a secondary endpoint of clinic spirometry (15). In the recent phase two *RELIEF* trial, the efficacy of pirfenidone was studied in CTD-ILD, fibrotic NSIP, fHP, and asbestos related progressive pulmonary fibrosis (e.g., ≥5% yearly FVC decline). Pirfenidone showed potential to slow decline in FVC in this population, though the trial was underpowered and stopped early due to poor enrollment (16).

Pirfenidone is administered three times per day (TID) on an escalating 2 week schedule, though dosing can be escalated at a slower rate if symptoms occur (17). Common side effects include nausea, vomiting, anorexia and rash (2, 14, 18). Gastrointestinal symptoms can be ameliorated with anti-emetics, antacids, and administration with adequate meals (14, 18). Elevations in ALT and AST can occur and LFTs should be monitored monthly for 6 months then quarterly (2, 14, 18). If side effects occur, dose reduction to six to eight capsules daily can be considered (2).

Tocilizumab, an inhibitor of interleukin-6 signaling, also has antifibrotic effects (19) and was recently shown to preserve FVC in systemic sclerosis related ILD as a secondary endpoint of the *focuSSced* trial (17), leading to its FDA approval for the treatment of scleroderma related ILD.

### Immunosuppression

Immunosuppression, while used for CTD and fHP, is harmful in IPF and should be avoided (20). Along with antigen avoidance, systemic oral corticosteroids are effective in non-fibrotic HP (21–23) and are recommended in fHP (24). More recent data, however, reveals corticosteroids may not improve mortality or lung function in fHP (23). In individuals with fHP on corticosteroids, transition to azathioprine or mycophenolate mofetil can improve side effects and stabilize lung function (25, 26). Leflunamide has recently been shown to modestly improve lung function and allow for corticosteroid cessation in chronic HP, but side effects were frequent and response in fibrotic disease was less robust (27). Overall, immunosuppression can be utilized in progressive fHP, though given the efficacy of nintedanib (9) their role is becoming less clear.

### Gastroesophageal Reflux Management

Gastroesophageal reflux is common in IPF (28) and connective tissue disease related ILD (29, 30) and may contribute to disease progression (2). Current guidelines recommend antacid therapy in IPF, though quality of evidence is low (20). In retrospective studies, antacids reduced exacerbations and lung function decline (31) and improved survival in IPF (32). However, a large post-hoc analysis showed no difference in mortality or FVC and more pulmonary infections with antacid use (33). Laparascopic anti-reflux surgery for high acid gastroesophageal reflux in IPF is generally safe but made no significant difference in outcomes in the *WRAP-IPF* trial (34). Larger scale randomized trials are needed to determine efficacy and safety of antacids and anti-reflux surgery in PF-ILD.

### Pulmonary Hypertension Therapies

World Health Organization (WHO) group 3 pulmonary hypertension (PH) (35) occurs frequently with ILD (36–38) and is associated with worse symptoms and exercise tolerance, earlier mortality, and increased need for supplemental oxygen (39, 40). Elevated right ventricular systolic pressure (RVSP) or right heart dysfunction on echocardiography should prompt investigation into comorbid hypoxemia or obstructive sleep apnea (OSA). Pulmonary vasodilators have historically shown mixed results in group 3 PH and current IPF guidelines recommend against use of sildenafil or endothelin receptor agonists (20). Riociguat increases mortality and should be avoided (41). Recently, however, inhaled treprostinil administered four times daily for 16 weeks improved exercise tolerance and N-terminal pro-brain natriuretic peptide (NT pro-BNP) levels in ILD associated PH (37), leading to its FDA approval as the first drug for ILD associated WHO group 3 PH.

## Non-Pharmacologic Therapies

### Supportive Care

Pneumococcal, influenza and COVID-19 vaccinations should be encouraged and administered to individuals with PF-ILD (8, 42). Intermittent hypoxia may contribute to pulmonary fibrosis development (43). Oxygen therapy should be prescribed for individuals with resting saturation or exertional desaturation ≤ 88% (2, 44) as it can improve exercise tolerance and exertional dyspnea (45, 46). Overnight oximetry should also be performed as maximal sleep desaturation can exceed exertional desaturation and worsen outcomes (47, 48). Screening for OSA should occur as it is common in IPF (49) and treatment may improve survival and quality of life (50). Pulmonary rehabilitation can improve exercise tolerance and quality of life for symptomatic individuals (2, 51, 52). If in-person facilities are unavailable, online rehabilitation may have similar efficacy (53). Anxiety and depression contribute to reduced quality of life and increased dyspnea and should be treated (54–57). Peer support programs can also add valuable psychosocial support (56).

### Lung Transplantation

ILD is the most common indication for lung transplantation, and transplantation has been shown to prolong survival (58) and improve symptoms (59) in PF-ILD (58–60). Post-transplant survival rates are improving yearly with most recent data revealing a 1 year survival rate of 88.8% and a 5 year survival rate of 59.2% (60). Survival rates amongst patients with ILD, however, are typically lower due to older age and comorbidities (60). For the appropriate patient, referral to a lung transplant center should be discussed early as the median wait time for transplantation once listed is around 3 months (60).

### Palliative Care

Despite current therapies, many patients with PF-ILD progress with significant end-of-life symptom burden. Disabling dyspnea can be treated safely with opiates or benzodiazepines (61, 62). Treatment of cough is challenging and should focus on optimization of other contributing disease processes (56). End of life care should be discussed early to avoid therapies that do not align with patient preferences. Early involvement of integrated palliative care may reduce end-of-life hospitalization and allow for more dignified deaths at home (63).

## Conclusions and Future Directions

Over the last 10 years there have been significant advances in the treatment of IPF and other PF-ILD. Nintedanib, pulmonary rehabilitation, and appropriate oxygen therapy should be utilized in individuals with progressive fibrotic disease. Lung transplantation should be considered in an interested, appropriate candidate. Inhaled treprostinil can be utilized in individuals with WHO group 3 PH and impaired functional status despite standard therapies. More research is needed to determine the efficacy of pirfenidone, immunosuppression and antacid therapy in all-cause and subsets of PF-ILD.

## Author Contributions

CC and LL contributed to conception and design of the study and drafted and critically revised the manuscript. All authors provided final approval of this version for submission.

## Conflict of Interest

The authors declare that the research was conducted in the absence of any commercial or financial relationships that could be construed as a potential conflict of interest.

## Publisher's Note

All claims expressed in this article are solely those of the authors and do not necessarily represent those of their affiliated organizations, or those of the publisher, the editors and the reviewers. Any product that may be evaluated in this article, or claim that may be made by its manufacturer, is not guaranteed or endorsed by the publisher.
